# Supplementary figures and images for: Electronic maternal and child health application usability, feasibility and acceptability among healthcare providers in Amhara region, Ethiopia
Source: PLOS Digit Health. 2024 May 13;3(5):e0000494. doi: 10.1371/journal.pdig.0000494 (PMC11090308; doi:10.1371/journal.pdig.0000494)

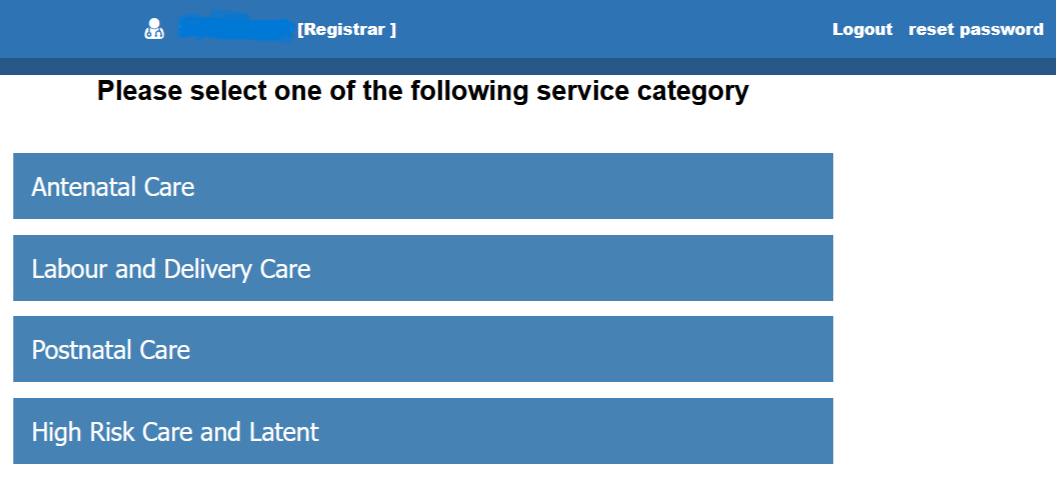

Supplement: S1 Fig — (PNG) [file pdig.0000494.s001.png]

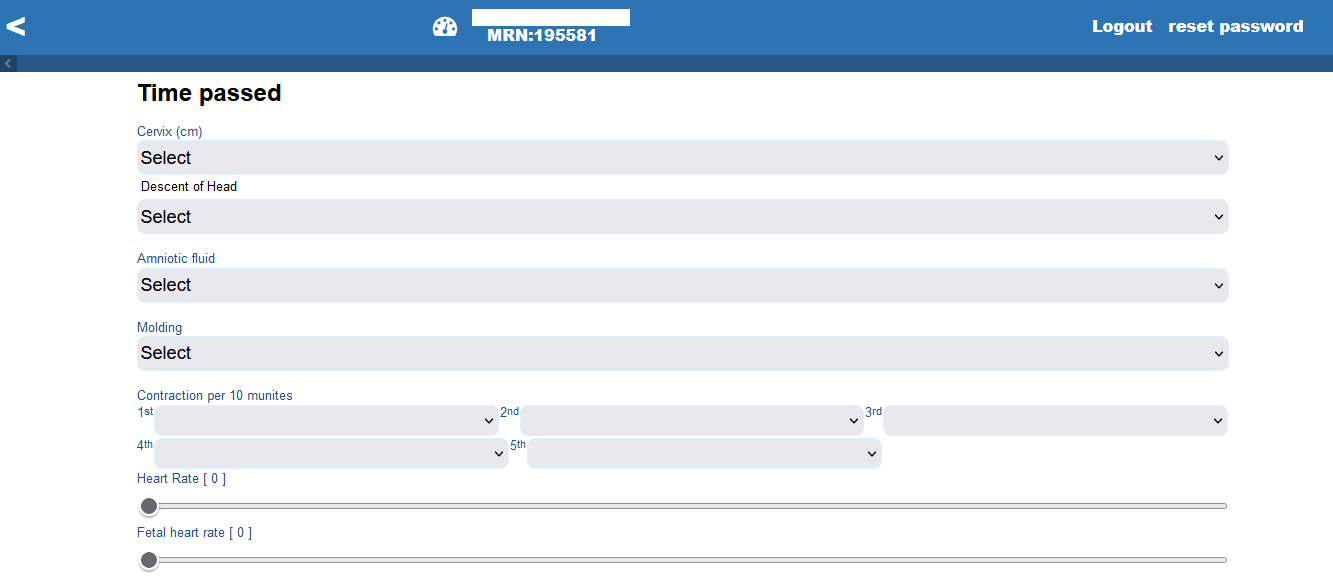

Supplement: S2 Fig — (PNG) [file pdig.0000494.s002.png]

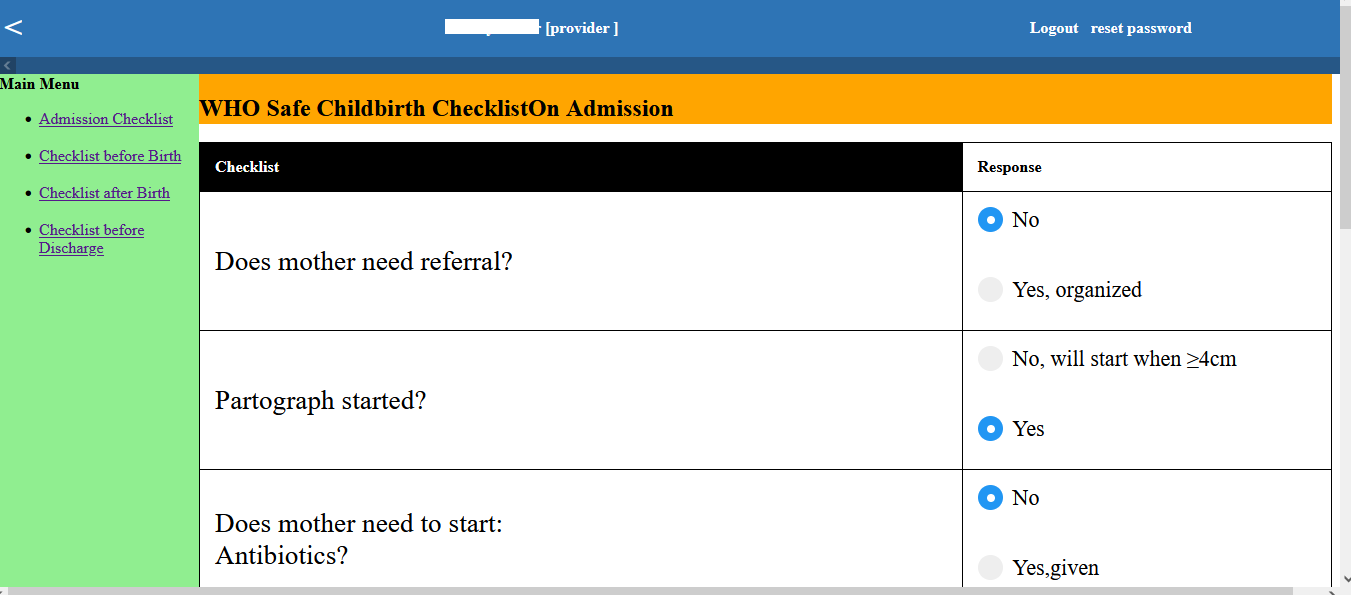

Supplement: S3 Fig — (PNG) [file pdig.0000494.s003.png]
