# Supplementary material for: Electronic maternal and child health application usability, feasibility and acceptability among healthcare providers in Amhara region, Ethiopia
Source: PLOS Digit Health. 2024 May 13;3(5):e0000494. doi: 10.1371/journal.pdig.0000494 (PMC11090308; doi:10.1371/journal.pdig.0000494)
Supplement: Attachment — Submitted filename: Response to preliminary revision request.docx [file pdig.0000494.s004.docx]

**Response to preliminary comments**

**Comment #1:** Your study includes live participants, but you have not included an Ethics Statement. Please include an Ethics Statement subsection to your Materials and Methods, to include:

i) (for human participants or donors) - A statement that formal consent was obtained (must state whether verbal/written) OR the reason consent was not obtained (e.g., anonymity).

**Response #1**: Thank you for your comment. I have included the “Ethics statement” subsection in the methods and materials section of the revised manuscript. Check line **217** to **222** for your reference.
