# Supplementary material for: Electronic maternal and child health application usability, feasibility and acceptability among healthcare providers in Amhara region, Ethiopia
Source: PLOS Digit Health. 2024 May 13;3(5):e0000494. doi: 10.1371/journal.pdig.0000494 (PMC11090308; doi:10.1371/journal.pdig.0000494)
Supplement: Attachment — Submitted filename: Response to reviewers.docx [file pdig.0000494.s005.docx]

**Response to reviewers**

**Response to reviewer #1**

**Comment #1:** The description of the study and tool is not clear. Is this a tool for medical providers or for patients or both? What does it do? what paper system does it replace? Was the paper tool validated before the development of the electronic tool? how many providers and institutions used it before?

***Response #1:*** *Thank you for the comment. We developed an eMCH tool, a quality improvement tool, to be used at point-of-care by healthcare providers. The eMCH is assumed to replace the paper-based maternal and newborn care to an electronic based-care with various added values. The added values include electronic decision support system, real-time data capturing, automatic drawing of graphs, which is a time-consuming task upon manual drawing, and provide reminders while entering out of range values. Moreover, it can ensure real-time data availability for decision at different levels in the health system (i.e., macro level (national, MoH, regional health bureau, policy), meso level (zonal health departments, districts health offices, healthcare facilities), and micro level (healthcare providers, health system managers) decisions) to improve maternal and newborn healthcare.*

*We conducted a small-scale piloting by deploying the tool in one hospital. During small-scale piloting, all healthcare providers such as medical doctors, midwifes who were working in the labour and delivery unit used the tool. Now, the tool was deployed in five healthcare facilities in Amhara region, Ethiopia.*

*In the revised submission, we included more detail description about the eMCH tool to make things clear for reader.*

**Comment #2:** Is there a central database where the data collected in the tool is stored for later review or is the data stored on the device in use? Are these mobile devices or desktop computers? What operating system is being used?

***Response #2:*** *Thank you for commenting. The data captured by the eMCH tool were stored in a server computer set as a central database. Thus, the data is available for later review and further analysis. We used window 10 operating system to run the computer.*

**Comment #3:** There are multiple spelling and grammatical errors. In the abstract it says 'thick-aloud' in at least two different places.

***Response #3:*** *Thank you for commenting. We corrected the editorial errors as suggested.*

**Comment #4:** The last sentence of the abstract is improper English: "Therefore, the tool should accommodate the recommended usability defects before deploying it in other healthcare settings." The correct term is not accommodated, nor defect. A more appropriate sentence would be: The errors and flaws of the tool should be fixed before deployment to other healthcare settings.

***Response #4:*** *Thank you for the insightful comment. We revised it accordingly.*

**Comment #5:** The word 'tenet' is used out of context. Not sure what the authors meant in the sentence. The word is improperly used.

***Response #5:*** *Thank you for the comment. The authors interest in the word ‘tenet’ as used in the sentence is to say ‘principle’.* *In the sentence, the core PHC tenet means the core PHC principle.*

**Comment #6:** The acronyms: ANC and PNC are used but not defined.

***Response #6:*** *Thank you for the comment. Now, we defined the acronyms at their first use in the manuscript.*

**Comment #7:** How was the data de-identified for analysis? Is it possible to roll back for further analysis?

***Response #7:*** *Thank you for the comment. Obviously, data to be released publicly or to be shared to other researchers has to be carefully de-identified. Hence, PII (personally identifiable information) variables like name, email, mobile number and address which has no role for analysis are dropped from the data before the analysis or sharing. We used pseudonymization where values of PII variables are replaced/masked by artificial identifiers. To enable roll back each identifier is unique and relationship with original/actual data is kept in a separate table. Whenever it necessitates, a person with desired role and expertise can generate the original values.*

**Comment #8:** Is there correlation between the age of the providers, their individual computer literacy and the results of the study regardless of the education level? Did older and less computer literate subjects make more errors?

***Response #8:*** *Thank you for the comments. It is obvious that computer literacy and age of users have effect on usability. Looking back at the data of this study, we found that the number of errors committed are correlated with computer literacy but not with age (Most errors are made by HCP at 1^st^ and 2^nd^ age ranges (Table 3)). This may be because the age range of the subjects is small.*

**Comment #9:** When the system has to be 'restarted' do they have to go back to the beginning and start over again or does the system save the data that is correct and the user can resume data entry where the error occurred?

***Response #9:*** *Thank you for your comment. The system has the capability to assist users start from where the restart was made. However, it depends on where the restart was made. For instance, in recording a patient data, if a user has entered majority of the data but a system restart was made before saving the data the user has to re-type the data. During the evaluation, we observed that most evaluators restarted from the beginning though they could continue from where they interrupted.*

**Comment #10:** Aside from data entry, is the tool and its contents used afterwards? or is it used only for tracking on the spot?

***Response #10:*** *The result of this study or this step, in general is needed to prepare the eMCH tool for next large scale pilot study. We upgraded the tool by considering all the usability flaws detected in the current study. Now, the tool is under large scale pilot study to show the effect of the eMCH tool in improving quality of maternal and newborn healthcare as well as its effect on positive pregnancy and child-birth outcomes. Therefore, the tool is under strict follow-up work for scale-up.*

**Comment #11:** Figure 1: what do the red question marks mean?

The delivery information is on top of the graph and difficult to read.

***Response #11:*** *The red question mark represents uterine contractions per 10 minute greater than 60. In the paper-partograph there is no space or room to entertain this value while actually uterine contractions per 10 minute greater than 60 is a possible condition as per the recommendation of experts in the field.*

*We placed the delivery information at the top of the partograph to make sure that the ePartograph align with the WHO recommended paper-partograph.*

**Comment #12:** Figure 2: the word efficiency is cut out

***Response #12:*** *We revised the chart and made it visible now.*

**Comment #13:** Figure 3: what is the difference between guidance and assistance? The figure does not stand on its own.

***Response #13:*** *thank you for your comment. The difference between guidance and assistance in this study is that in the case of guidance an eMCH-user required thorough guide or action from facilitators to perform the tasks given while in the case of assistance an eMCH-user required only a limited help from facilitators to perform the tasks given. In the case of guidance, the facilitators played a critical role for a user to accomplish the tasks using the eMCH tool. In the current submission, we revised figure 4 caption to include legends.*

**Comment #14:** I recommend correcting the spelling and grammatical errors.

***Response #14:*** *We revised the manuscript to address editorial concerns.*

**Comment #15:** Add a section with a brief description of the tool so the reader can better understand what the tool is about. Description of why is an electronic tool better than a paper tool.

***Response #15:*** *We included additional description in the section dedicated for eMCH tool description.*

**Comment #16:** What is the return on investment of the electronic tool?

***Response #16:*** *The eMCH tool we developed is a quality improvement tool to be deployed in healthcare facilities serving maternal and newborn care (ANC, Delivery and PNC). For instance, WHO recommend partograph use to monitor labour progress for every labouring woman. Low rate of use, incomplete recording of parameters, and improper completion of partograph were a problem in Ethiopia. We hypothesized that digitalizing the partograph have added values to improve the uptake and quality of care, which in turn have a positive impact on maternal and child survival. Besides, one of the objectives we planned to address in our pilot study is cost evaluation, cost analysis or incremental cost associated with deployment of eMCH tool in five public healthcare facilities in Amhara region, Ethiopia.*

**Comment #17:** Compare age-computer literacy and usability results. The variables are there but there are no correlations between the variables.

***Response #17:*** *Thank you for your comment. On top our response for comment #8, during the screening stage, we have screened out HCP whose computer skills are below basic. This is because their skill is so low from standard that they won’t be able to complete any the tasks despite the guidance. The variables are presented for completeness of demographic data.*

**Comment #18:** An English editor is necessary to make sure the correction of the grammatical errors reflects what the authors want to describe.

***Response #18:*** *thank you for the comment. We thoroughly revised for editorial issues. Besides, we applied online grammar checking tools such as Grammarly, and quill bot.*

**Response to reviewer #2**

**Comment #1:** I would like to commend how the different parameters were explained in detail. However, some grammatical errors were seen in the Method and Materials section. It would also be better if the statistical analysis used was explained.

***Response #1:*** *Thank you for commenting this work. We considered revising the entire manuscript to improve the grammatical errors as well as the description given on statistical analysis.*
