# Supplementary material for: Electronic maternal and child health application usability, feasibility and acceptability among healthcare providers in Amhara region, Ethiopia
Source: PLOS Digit Health. 2024 May 13;3(5):e0000494. doi: 10.1371/journal.pdig.0000494 (PMC11090308; doi:10.1371/journal.pdig.0000494)
Supplement: Attachment — Submitted filename: Response to reviewers.docx [file pdig.0000494.s006.docx]

**Response to reviewers**

**Response to reviewer #3**

**Comment #1:** How do you define 'error' and 'restarts'?

***Response #1:*** *Error refers to users’ activities that are not relevant or necessary to accomplish a given task on eMCH application, which include clicking wrong link or unclickable image, clicking save/complete and other buttons before entering/selecting all mandatory fields, entering/selecting wrong values, and double clicks. Restarts refer to frequency of reloading of eMCH application to accomplish a given task using the referent application.*

**Comment #2:** Why have you used KW test and not One-way ANOVA to compare samples?

***Response #2:*** *Thank you for your comment. We preferred to use Kruskal-Wallis test because KW test is non-parametric test while the ANOVA is a parametric test. ANOVA assumes normality of the data. Since the number of participants involved in this evaluation are small, the data didn’t meet normality assumption to use one way ANOVA test. We already explained it in the limitation section of the manuscript.*

**Comment #3:** Please describe 'think aloud protocol'

***Response #3:*** *Thank you for your comment. We have addressed this comment in the revised submission.*

**Comment #4:** How do you define 'integrative, interoperable and vendor-neutral digital health platform'?

***Response #4:*** *Thank you for your comments. Vendor-neutral digital health platform refers to a platform that doesn’t involves locking data and it liberates data from the proprietary ownership. While preserving security and privacy, vendor-neutral digital health platform provides health care providers an access to patient data making the data obtainable when and where they need it. Interoperability on the other hand is the ability of the system to work with other related systems by exchanging data. So, integrative, interoperable and vendor-neutral digital health platform is a digital health platform that enables open data access and has the capability to integrate with other systems to achieve interoperability.*

**Comment #5:** Please avoid 'we', 'our', 'us' in the text.

***Response #5:*** *Thank for the insight, we have gone through the manuscript and fixed the issues raised by the reviewer.*

**Comment #6:** Show few screenshots of the eMCH tool for generating more relevance.

***Response #6:*** *Thank you for your comments. We provided additional images as supporting information.*

**Comment #7:** Recommendations:

*1. Chattopadhyay, S. Shinha, P. “Understanding Factors Impacting COVID Vaccination in India: A Preliminary Report”. Quantum Journal of Medical and Health Sciences, (2021), 1(3): 18-31.*

*2. Chattopadhyay S., Daneshgar F. – “An Awareness Net Collaborative Model for Schizophrenia Management”, International Journal of Advanced Intelligence Paradigms (2013); 5(3): 217-232.*

…

*14. Li J, Land L, Chattopadhyay S., Ray P. – 'An Approach for E-health System Assessment & Specification'. In proceedings of 10th IEEE Intl. Conf. on e-Health Networking, Applications and Service (HEALTHCOM 2008), pp. 157-162, Singapore (2008).*

Please note some of these research papers showcase early intervening models of eHealth and helped building current pillars of eHealth solutions/tools and required to be cited.

***Response #7:*** *We applied our concerted efforts to review the list of references given by the reviewer as far as we were not encounter open access restriction. The review was framed based on relevance to the current study. For those references we found relevant to full-text review we were unable to access the papers because of open access restrictions.*
